# Garlic-derived S-allylmercaptocysteine is a hepato-protective agent in non-alcoholic fatty liver disease in vivo animal model

**DOI:** 10.1007/s00394-012-0301-0

**Published:** 2012-01-26

**Authors:** Jia Xiao, Yick Pang Ching, Emily C. Liong, Amin A. Nanji, Man Lung Fung, George L. Tipoe

**Affiliations:** 1 Department of Anatomy, Li Ka Shing Faculty of Medicine, The University of Hong Kong, L1-41, Laboratory Block, 21 Sassoon Road, Pokfulam, Hong Kong, SAR; 2 Department of Physiology, Li Ka Shing Faculty of Medicine, The University of Hong Kong, Pokfulam, Hong Kong; 3 Department of Pathology and Laboratory Medicine, Faculty of Medicine, Dalhousie University, Halifax, NS Canada

**Keywords:** S-allylmercaptocysteine, NAFLD, Fibrosis, Oxidative stress, Inflammation

## Abstract

**Purpose:**

To investigate the hepato-protective properties and underlying mechanisms of SAMC in a non-alcoholic fatty liver disease (NAFLD) rat model.

**Methods:**

Female rats were fed with a diet comprising highly unsaturated fat diet (30% fish oil) for 8 weeks to develop NAFLD with or without an intraperitoneal injection of 200 mg/kg SAMC three times per week. After euthanasia, blood and liver samples of rats were collected for histological and biochemical analyses.

**Results:**

Co-treatment of SAMC attenuated NAFLD-induced liver injury, fat accumulation, collagen formation and free fatty acids (FFAs). At the molecular level, SAMC decreased the lipogenesis marker and restored the lipolysis marker. SAMC also reduced the expression levels of pro-fibrogenic factors and diminished liver oxidative stress partly through the inhibition in the activity of cytochrome P450 2E1-dependent pathway. NAFLD-induced inflammation was also partially mitigated by SAMC treatment via reduction in the pro-inflammatory mediators, chemokines and suppressor of cytokine signaling. The protective effect of SAMC is also shown partly through the restoration of altered phosphorylation status of FFAs-dependent MAP kinase pathways and diminished in the nuclear transcription factors (NF-κB and AP-1) activity during NAFLD development.

**Conclusions:**

SAMC is a novel hepato-protective agent against NAFLD caused by abnormal liver functions. Garlic or garlic derivatives could be considered as a potent food supplement in the prevention of fatty liver disease.

**Electronic supplementary material:**

The online version of this article (doi:10.1007/s00394-012-0301-0) contains supplementary material, which is available to authorized users.

## Introduction

Non-alcoholic fatty liver disease (NAFLD) is one of the forms of chronic liver diseases characterized by hepatic fibrosis, oxidative stress and inflammation. According to the histological classifications in clinical diagnosis, the so-called NAFLD ranges from fat accumulation in the hepatocytes without obvious fibrosis or inflammation to severe hepatosteatosis that may progress to cirrhosis and hepatocellular carcinoma [1]. It is an emerging hepatic disease with an estimated prevalence of 20–40% in Western countries [2]. The molecular mechanism for the initiation and progression of NAFLD is summarized by a “multi-hit” theory, where imbalanced lipid metabolism and insulin resistance is considered as the first hit to the liver. When insulin resistance occurs, liver becomes more vulnerable to hyperinsulinemia-induced “multi-hit” events involving the release of reactive oxygen species (ROS), pro-fibrogenic factors and pro-inflammatory mediators from impaired organelles. Subsequently, activated hepatic stellate cells induce excessive accumulation of extracellular matrix proteins, such as collagen, to cause fibrosis with concomitant necroinflammation and cell death [3, 4].

S-allylmercaptocysteine (SAMC) is a water-soluble sulfur compound present in aged garlic extract. Previous studies have shown that SAMC is effective in inhibiting cell growth and promoting apoptosis in several cancer cell lines [5–7]. Moreover, SAMC is also shown to play antioxidative and anti-nitrosative stress roles both in vitro and in vivo [8]. The hepato-protective effect of SAMC is exhibited in an acetaminophen-induced liver injury model and a carbon tetrachloride-induced acute injury model where SAMC inhibits the activity of cytochrome P450 2E1 (CYP2E1) and reduces oxidative stress released by chemical intoxication [9–11]. However, the protective effects and underlying mechanisms of SAMC in NAFLD remain unknown. In this study, we demonstrated that administration of SAMC in a high-fat diet voluntarily and orally fed rat model effectively attenuated NAFLD-induced hepatic injury, fibrosis, oxidative stress and inflammation partly through free fatty acids (FFAs)-dependent pathways and nuclear transcription factors.

## Materials and methods

### Reagents

SAMC pure powder was kindly given by Dr. Patrick M. T. Ling (Queensland University of Technology, Australia) and originally from Wakunaga Co. Ltd (Osaka, Japan). The purity of the SAMC powder is more than 95% by HPLC analysis. It does not contain any other garlic compound such as SAC or allicin. The powder was dissolved in phosphate-buffered saline containing 10% l-dextrose and 1% gum Arabic (w/v) at pH 4.5. Murine anti-nitrotyrosine monoclonal antibody was bought from Zymed (San Francisco, CA, USA). Rabbit anti-CYP2E1 polyclonal antibody was purchased from Millipore (Billerica, MA, USA). Antibody against phosphorylated ERK1/2 at Tyr204 (p-ERK) was bought from Santa Cruz Biotechnology (Santa Cruz, CA, USA). Antibody of total c-Jun was obtained from BD Biosciences (San Jose, CA, USA). Antibodies of SOCS3, phosphorylated c-Jun at Ser63 (p-cJun), phosphorylated p38 MAPK at Thr180/Tyr182 (p-MAPK), total p38 MAPK, phosphorylated IκBα at Ser32 (p- IκBα), total IκBα, phosphorylated MEK1/2 at Ser217/221 (p-MEK), total MEK1/2, total ERK1/2, phosphorylated JNK at Thr183/Tyr185 (p-JNK), and total JNK were purchased from Cell Signaling (Danvers, MA, USA).

### Animal experiments

Eight-week healthy female SD rats with body weight ranging from 180 to 200 g were purchased from the Laboratory Animal Unit (LAU), The University of Hong Kong. Rats were kept under standard conditions for 3 days before the start of the experiment with free access to animal chow and tap water. The animals were divided into four groups (*n* = 7 per group) namely: (1) control group; (2) NAFLD group; (3) SAMC treatment only group (200 mg/kg in solvent; intraperitoneal injection; three times per week); and (4) NAFLD and SAMC co-treatment group. The development of NAFLD in rats, including the recipe and preparation protocols of diet, was performed based on our previously described voluntary oral feeding NAFLD animal model [12, 13]. Instead of oral administration or in a dietary form, SAMC was intraperitoneally injected to avoid possible degradation prior to absorption through the gastrointestinal tract (GIT). The diet consists of 9.3 g AIN-93MX (Dyets incorporation, Bethlehem, PA), 2.6 g AIN-93VX (Dyets), 0.5 g choline bitatrate (Dyets), 1.1 g DL-methione (Bio-serv, Frenchtown, NJ), 57.5 g lactalbulmine hydrolysate (Bio-serv), 117.5 g dextrose (Dyets), 36.6 ml fish oil (Sigma) and 4.5 g suspending agent K (Bio-serv) in per 1,000 ml volume. Regular chow for rat (PicoLab^®^ Rodent Diet 20) was purchased from LabDiet (LabDiet, Brentwood, MO, USA). The calories of regular chow were provided by 25% from protein, 13% from fat and 62% from carbohydrates, while the calories of high-fat diet were provided by 35% from protein, 30% from fat and 35% from carbohydrates. The optimum dosage of SAMC was previously shown to be effective in protecting the liver from hepatic oxidative stress in mice [14]. After 8 weeks, the rats were euthanized by an overdose of anesthesia (intraperitoneal injection of 150 mg/kg pentobarbitone sodium) according to the protocols approved by the Committee of Animal Use for Research and Teaching at The University of Hong Kong. The Laboratory Animal Unit of the University of Hong Kong is fully accredited by the Association for Assessment and Accreditation of Laboratory Animal Care International (AAALAC international). Blood and liver samples were collected for further analysis.

### Processing of tissue and blood samples

Serum was collected by centrifugation of whole blood sample at 1,000×*g* for 10 min at 4 °C and stored at −80 °C. Liver tissue samples were fixed in 10% phosphate-buffered formalin, processed for histology and embedded in paraffin blocks. Five-micrometer tissue sections were cut and stained with hematoxylin and eosin (H&E) or Sirius Red for histological analysis under LEICA Qwin Image Analyser (Leica Microsystems Ltd., Milton Keynes, UK). The percentage area of the total amount of collagen (7 sections for each group) was quantified by the sum areas of Sirius Red positive staining divided by the reference field multiplied by 100. The severity of NAFLD of each group was assessed by using the NAFLD activity scoring (NAS) system as previously described [15]. The mean NAS score was calculated for each experimental group.

### Serum alanine aminotransferase (ALT) assay

To evaluate the hepatic injury at the enzymatic level, serum ALT level was measured by using ALT (SGPT) reagent set (Teco diagnostics, Anaheim, CA, USA) according to manufacturer’s instructions.

### Free fatty acids (FFAs) assay

To study the effect of SAMC on lipid metabolism, serum FFAs level of each rat was measured by using Cayman free fatty acids assay kit (Cayman chemical, Ann Arbor, MI, USA), and the final results were expressed as μM in the serum.

### Measurement of malondialdehyde (MDA) level

To investigate the possible effects of SAMC on hepatic lipid peroxidation, levels of the end product of lipid peroxidation (MDA) in all liver tissue samples were determined by using a Bioxytech LPO-586™ kit (Oxis Research, Portland, OR, USA). The reaction product was measured spectrophotometrically at 586 nm. Standard curves were constructed using 1,1,3,3-tetraethoxypropane as a standard. The MDA levels were normalized with corresponding protein amounts determined by a Bio-Rad Protein Assay Kit (Bio-Rad, Hercules, CA, USA) and expressed as percentage against the control level.

### RNA extraction and reverse transcription-quantitative polymerase chain reaction

Total RNA of each rat was extracted from the liver sample by using illustra™ RNAspin mini kit (GE healthcare, UK). The preparation of the first-strand cDNA was conducted following the instruction of the SuperScript™ First-Strand Synthesis System (Invitrogen, Calsbad, CA, USA).

The mRNA expression levels of sterol regulatory element binding protein-1c (SREBP1c), adiponectin, tumor necrosis factor-alpha (TNF-α), interleukin-1 beta (IL-1β), inducible nitric oxide synthase (iNOS), cyclooxygenase-2 (COX-2), glutathione peroxidase (GPx), catalase (CAT), monocyte chemoattractant protein-1 (MCP-1), macrophage inflammatory protein-2 (MIP-2), KC (murine IL-8 ortholog), transforming growth factor-beta1 (TGF-β_1_), procollagen-1 (PC-1), alpha-smooth muscle actin (α-SMA) and suppressor of cytokine signaling-3 (SOCS-3) were measured by Takara SYBR premix Taq quantitative PCR system (Takara Bio Inc, Shiga, Japan) and in MyiQ2 real-time PCR machine (Bio-Rad). The primer sequences and annealing temperatures used in those Q-PCR are listed in Table 1. All primers were designed by using Primer Premier 5 (Premier Biosoft, Palo Alto, CA, USA) software with specificity validation. PCR efficiency of each primer pair was tested. Parallel amplification of glyceraldehyde-3-phosphate dehydrogenase (GAPDH) was used as the internal control. Relative quantification was done by using the 2^−ΔΔCt^ method. The relative expression of the specific gene to the internal control was obtained and then expressed as a percentage of the control value in the figures. All quantitative PCR procedures including the design of primers, validation of PCR environment and quantification methods were performed according the MIQE guideline [16].

**Table 1:** Primer sequences and annealing temperatures for quantitative PCR

Target gene	Direction	Primer sequence (5′–3′)	A. Temp. (°C)
SREBP1c	Forward	GGAGCCATGGATTGCACATT	58
	Reverse	GCTTCCAGAGAGGAGCCCAG	
Adiponectin	Forward	TAAGGGTGACCCAGGAGATG	58
	Reverse	GGA ACATTGGGGACAGTGAC	
TNF-α	Forward	ATGAGCACAGAAAGCATGATC	62
	Reverse	TACAGGCTTGTCACTCGAATT	
IL-1β	Forward	CACCTCTCAAGCAGAGCACAG	60
	Reverse	GGGTTCCATGGTGAAGTCAAC	
iNOS	Forward	CATTGGAAGTGAAGCGTTTCG	58
	Reverse	CAGCTGGGCTGTACAAACCTT	
COX-2	Forward	TGTATGCTACCATCTGGCTTCGG	58
	Reverse	GTTTGGAACAGTCGCTCGTCATC	
GPx	Forward	TCCACCGTGTATGCCTTCTCC	58
	Reverse	CCTGCTGTATCTGCGCACTGGA	
CAT	Forward	GAGGCAGTGTACTGCAAGTTCC	58
	Reverse	GGGACAGTTCACAGGTATCTGC	
MCP-1	Forward	ACCAGCCAACTCTCACTGAAGC	60
	Reverse	CAGAATTGCTTGAGGTGGTTGTG	
MIP-2	Forward	AGTGAACTGCGCTCTCAATG	55
	Reverse	CTTTGGTTCTTCCGTTGAGG	
KC	Forward	CTGTCAGTGCCTGCAGACCA	56
	Reverse	CCAAGGGAGCTTCAGGGTCA	
IL-6	Forward	CCGGAGAGGAGACTTCACAG	60
	Reverse	GGAAATTGGGGTAGGAAGGA	
TGF-β_1_	Forward	CTTCAGCTCCACAGAGAAGAACTGC	60
	Reverse	CACGATCATGTTGGACAACTGCTCC	
PC-1	Forward	TGCCGTGACCTCAAGATGTGCC	60
	Reverse	CATCCACAAGCGTGCTGTAGGTG	
α-SMA	Forward	CTGGAGAAGAGCTACGAACTGC	55
	Reverse	CTGATCCACATCTGCTGGAAGG	
SOCS-3	Forward	CCTCCAGCATCTTTGTCGGAAGAC	58
	Reverse	TACTGGTCCAGGAACTCCCGAATG	
GAPDH	Forward	CCTTCATTGACCTCAACTACATGGT	55
	Reverse	TCATTGTCATACCAGGAAATGAGCT	

*A. Temp.* annealing temperature

### Western blot analysis

Cytosolic and nuclear protein extraction of rat liver was conducted using NE-PER protein extraction system (Pierce Biotechnology, Rockford, IL, USA) with the addition of Halt phosphatase inhibitor cocktail (Pierce). Before Western blot, protein was diluted and mixed with 2× sample buffer (0.1 M Tris–HCl, pH 6.8, 20% glycerol, 4% sodium dodecyl sulfate, 0.2% Bromophenol Blue, 5.25% β-mercaptoethanol). The mixture was denatured at 99 °C for 5 min and followed by electrophoresis in a 10% polyacrylamide gel. The protein was then transferred to an Immun-Blot™ PVDF Membrane (Bio-Rad) in a TE series transfer electrophoresis unit (Hoefer Inc., Holliston, MA, USA). The membrane was then incubated in blocking buffer (5% non-fat milk powder in TBST, 100 mM Tris–HCl, pH 7.5, 0.9% NaCl, 0.1% Tween 20) for 1 h followed by incubation with different primary antibodies in TBST overnight at 4 °C with gentle agitation. On the following day, the membrane was washed with TBST and incubated with appropriate secondary antibodies for 2 h at room temperature. Beta-actin was used as the internal control. After washing off the unbound antibody with TBST, the expression of the antibody-linked protein was determined by an ECL™ Western Blotting Detection Reagents (GE Healthcare). The optical density of the bands was measured and quantified by ImageJ software (National Institute of Health, MD). The ratio of the optical density of the protein product to the internal control was obtained and was expressed as a percentage of the control value in the figures.

### Enzyme-linked immunosorbent assay (ELISA) measurement

To correlate the mRNA expression with protein expression of selected target genes in the liver, ELISA measurements of TNF-α, IL-1β, MCP-1 and TGF-β_1_ were performed by using corresponding ELISA development kits from PeproTech (PeproTech Inc., Rocky Hill, NJ, USA) according to user instructions.

### DNA-binding activity of nuclear factor-κB (NF-κB) and activator protein-1 (AP-1)

Determination of NF-κB and AP-1 are the major transcription factors in the regulation of inflammation and oxidative stress. The DNA-binding activity of these factors was performed by electrophoretic mobility shift assay (EMSA) using the Gel-Shift Assay Systems from Promega (Promega, Madison, WI, USA). Briefly, the phosphorylated and purified consensus NF-κB or AP-1 oligonucleotides (Promega) with ^32^P labeling was mixed with 24 μg of nuclear protein extract and 10× Gel-Shift Binding Buffer (200 mm Tris–HCl, pH 7.8, 1 m NaCl, 50 mm MgCl_2_, 10 mm EDTA and 50 mm dithiothreitol). The mixture was incubated at room temperature for 20 min prior to electrophoresis on a 4% non-denaturing polyacrylamide gel. Signals on exposed X-ray films were quantified using laser scanning densitometry. Specificity of NF-κB binding was confirmed by competition assays and the ability of a specific antibody to supershift protein-DNA complexes. In the competition assay, the addition of 100-fold excess of unlabeled competitor consensus oligonucleotide prevented binding. To confirm the specificity of NF-κB or AP-1 binding, supershift experiments with p50/p60 antibody addition or c-Jun antibody addition were performed, respectively.

### Statistical analysis

Data from each group were expressed as means ± SEM. Statistical comparison between groups was done using the Kruskal–Wallis test followed by Dunn’s post hoc test to detect differences in all groups. A *p* < 0.05 was considered to be statistically significant (Prism 5.0, Graphpad software, Inc., San Diego, CA, USA).

## Results

**Fig. 1:**
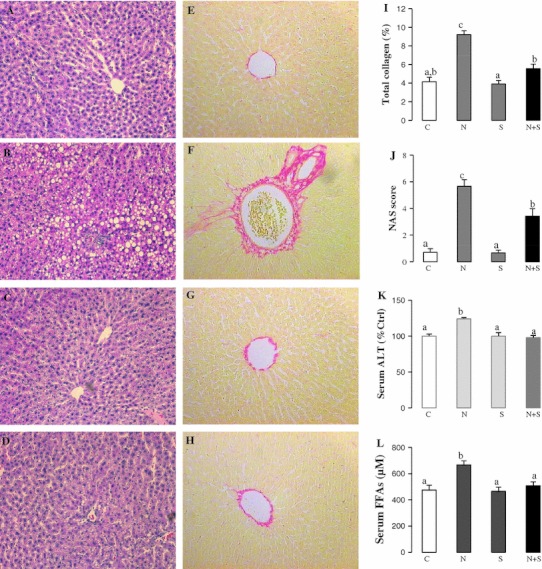
Ameliorative effects of SAMC on liver injury, collagen accumulation and excessive free fatty acids. Liver H&E staining of **a** control group, **b** NAFLD group, **c** SAMC group and **d** NAFLD+SAMC group. Liver Sirius Red staining of **e** control group, **f** NAFLD group, **g** SAMC group and **h** NAFLD+SAMC group. **i** Percentage of total collagen of liver with Sirius Red staining, **j** NAS scoring of liver H&E staining, **k** serum ALT level and **l** serum free fatty acids (FFAs) level of each group. Magnification: ×200. Data presented are expressed as Mean ± SEM (*n* = 7) and experimental groups marked by *different letters* represented significant differences between groups at *p* < 0.05 (Kruskal–Wallis test followed by Dunn’s post hoc test). *C* control, *N* NAFLD, *S* SAMC; *N*+*S* NAFLD+SAMC

Since NAFLD is characterized by liver injury and fibrosis, as well as increased FFAs in the blood, we measured the effects of SAMC administration along with the NAFLD development on liver histology, serum ALT and FFAs levels. NAFLD induced cellular necrosis, inflammation and collagen accumulation surrounding the centrilobular veins of the liver (Fig. 1b, f). Co-treatment of SAMC markedly attenuated the hepatic injury, steatosis, inflammation and fibrosis without affecting the normal liver cells (Fig. 1d, h). The percentage of collagen distribution of the liver also showed a marked reduction in the amount of collagen in SAMC co-treatment rats, which supported the anti-fibrotic effects of the SAMC treatment (Fig. 1i). The NAS score of NAFLD rats was high (5.67 ± 0.49), indicating severe steatohepatitis after the induction from high-fat diet. Co-treatment with SAMC significantly reduced the NAS score to a “borderline of NASH” level (3.42 ± 0.57; Fig. 1j) [15]. Moreover, serum ALT-indicated lesser injury and FFAs induced by NAFLD were significantly down-regulated to control levels by SAMC co-treatment (Fig. 1k, l).

**Fig. 2:**
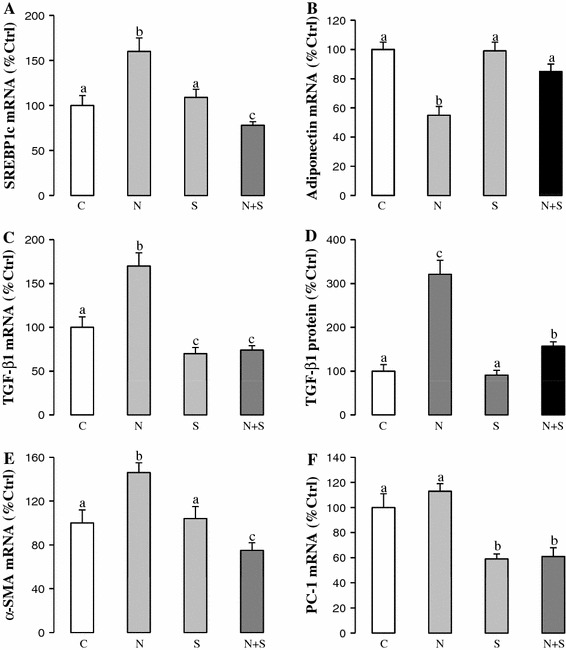
SAMC restored lipid metabolism status and reduced fibrogenic factors induced by NAFLD. Expression levels of **a** SREBP1c mRNA, **b** adiponectin mRNA, **c** TGF-β_1_ mRNA, **d** TGF-β_1_ protein, **e** α-SMA mRNA and **f** PC-1 mRNA were measured in rat liver after NAFLD development with or without co-treatment of SAMC by quantitative PCR or ELISA. Data presented are expressed as Mean ± SEM (*n* = 7) and experimental groups marked by *different letters* represented significant differences between groups at *p* < 0.05 (Kruskal–Wallis test followed by Dunn’s post hoc test). *C* control, *N* NAFLD, *S* SAMC, *N*+*S* NAFLD+SAMC

Increased expression of lipogenesis marker gene (SREBP1c) was observed in the NAFLD rats, whereas the expression of lipolysis marker gene (adiponectin) was reduced, suggesting an on-going process of hepatic lipid accumulation. Addition of SAMC reversed such trend by down-regulating SREBP1c and up-regulating adiponectin (Fig. 2a, b).

To further study the effects of SAMC on hepatic fibrosis, expression change of key pro-fibrogenic mediators including TGF-β_1_, α-SMA and PC-1 were measured by quantitative PCR or ELISA. NAFLD induced the expression of TGF-β_1_ and α-SMA at both transcriptional and translational levels. When SAMC was co-treated, these genes were down-regulated and reduced to levels almost comparable to their corresponding controls (Fig. 2c–e). The PC-1 mRNA expression in SAMC co-treatment group also showed significant reduction when compared with NAFLD rats (Fig. 2f).

**Fig. 3:**
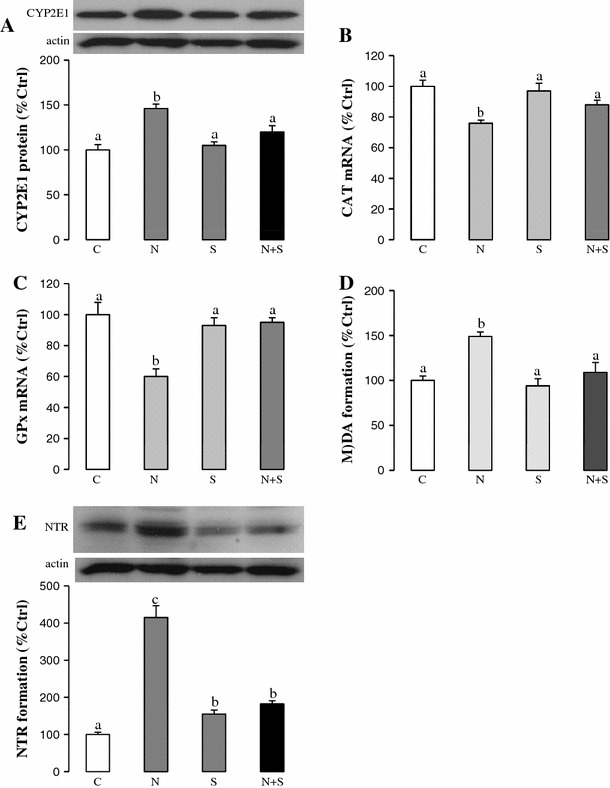
Ameliorating effects of SAMC on NAFLD-induced oxidative stress. Expression of **a** cytochrome P450 2E1 (CYP2E1) protein, **b** CAT mRNA, **c** GPx mRNA, **d** MDA formation and **e** nitrotyrosine formation were measured in rat liver after NAFLD development with or without co-treatment of SAMC by quantitative PCR or Western blot. Data presented are expressed as Mean ± SEM (*n* = 7) and experimental groups marked by *different letters* represented significant differences between groups at *p* < 0.05 (Kruskal–Wallis test followed by Dunn’s post hoc test). *C* control, *N* NAFLD, *S* SAMC, *N*+*S* NAFLD+SAMC

To investigate the possible protective function of SAMC on increased oxidative stress that plays a critical role on the further development of steatohepatitis, key oxidative stress markers during NAFLD were measured. Up-regulated expression of CYP2E1 during NAFLD was counteracted by SAMC co-treatment (Fig. 3a). Administration of SAMC also restored the mRNA expression of antioxidant enzymes, CAT and GPx, which were down-regulated during NAFLD (Fig. 3b, c). Co-treatment of SAMC also reduced the formation of MDA and nitrotyrosine during NAFLD development without disturbing their basal levels (Fig. 3d, e).

**Fig. 4:**
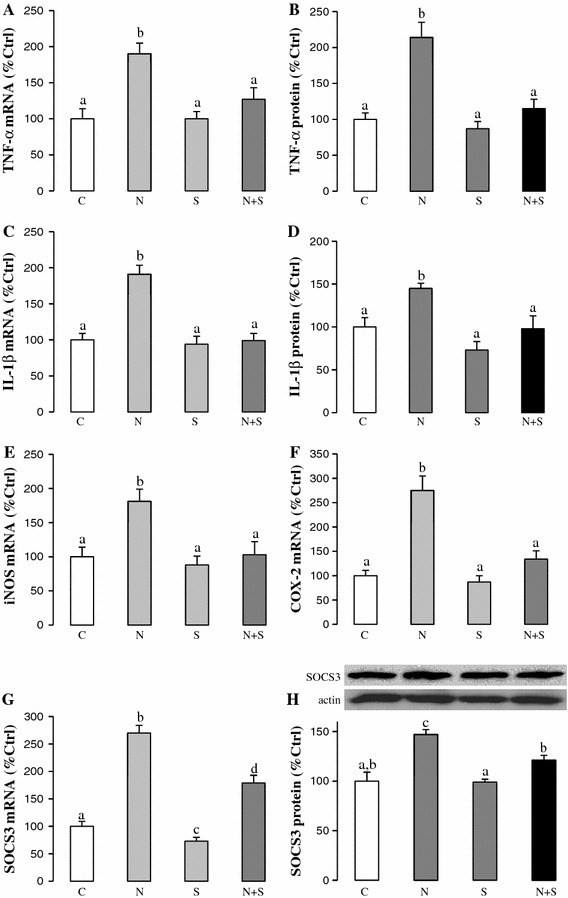
Anti-inflammation properties of SAMC during NAFLD development. Expression of **a** TNF-α mRNA, **b** TNF-α protein, **c** IL-1β mRNA, **d** IL-1β protein, **e** iNOS mRNA, **f** COX-2 mRNA, **g** SOCS3 mRNA and **h** SOCS3 protein were measured in rat liver after NAFLD development with or without co-treatment of SAMC by quantitative PCR or ELISA/Western blot. Data presented are expressed as Mean ± SEM (*n* = 7) and experimental groups marked by *different letters* represented significant differences between groups at *p* < 0.05 (Kruskal–Wallis test followed by Dunn’s post hoc test). *C* control, *N* NAFLD, *S* SAMC, *N*+*S* NAFLD+SAMC

**Fig. 5:**
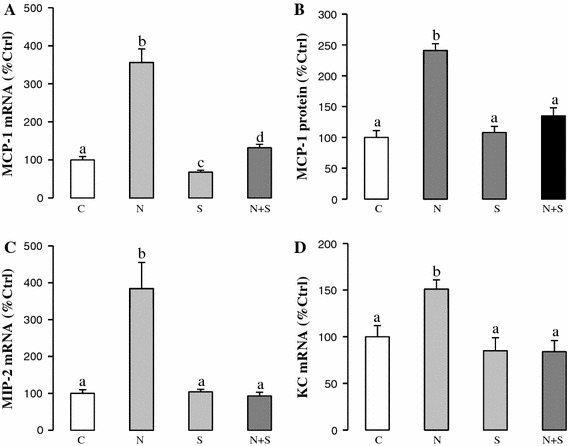
Co-treatment of SAMC reduced NAFLD-induced chemokine expression. Expression level of **a** MCP-1 mRNA, **b** MCP-1 protein, **c** MIP-2 mRNA and **d** KC mRNA were measured in rat liver after NAFLD development with or without co-treatment of SAMC by quantitative PCR or ELISA. Data presented are expressed as Mean ± SEM (*n* = 7), and experimental groups marked by *different letters* represented significant differences between groups at *p* < 0.05 (Kruskal–Wallis test followed by Dunn’s post hoc test). *C* control, *N* NAFLD, *S* SAMC, *N*+*S* NAFLD+SAMC

Inflammation and subsequent chemoattraction of cell migration to the liver are critical for the development and progression of NAFLD [17]. We measured several inflammatory mediators and chemokines in NAFLD rats with or without SAMC co-treatment. Consistent with previous reports, NAFLD induced expressions of pro-inflammatory mediators (TNF-α, IL-1β, iNOS, COX-2) (Fig. 4a–f) and chemokines (MCP-1, MIP-2, KC) (Fig. 5a–d) at both mRNA and protein levels. Administration of SAMC during NAFLD development significantly attenuated the inductions of the expression levels of these inflammatory mediators when compared to the controls, with values near to control levels. As the negative regulator of the inflammatory response, both mRNA and protein expressions of SOCS3 were increased during NAFLD development. Co-treatment of SAMC also abolished the induced SOCS3 expression (Fig. 4g, h).

**Fig. 6:**
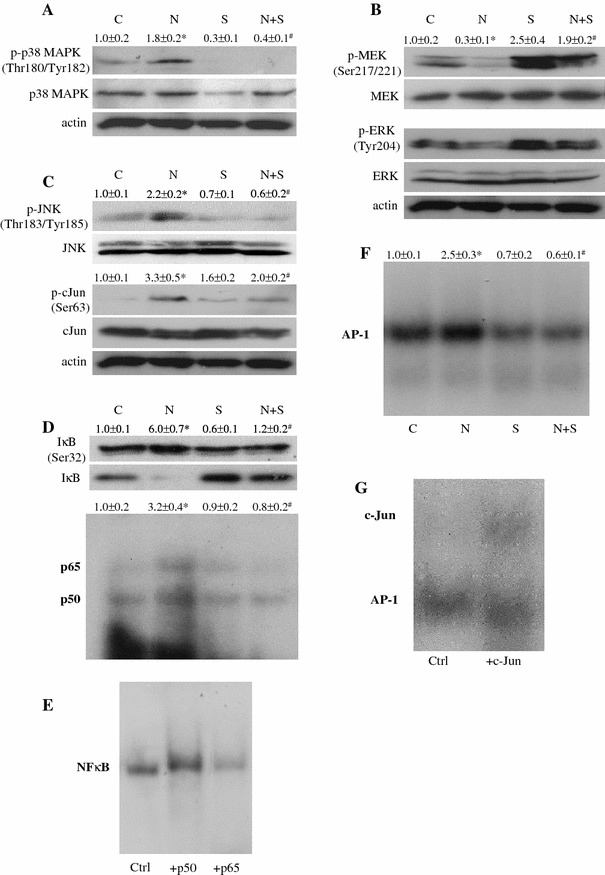
The effects of SAMC on signaling molecules and transcription factors, which mediate NAFLD-induced insulin resistance and liver injury. Representative results of the phosphorylation status and total expression of **a** p38 MAPK, **b** MEK1/2–ERK1/2 and **c** JNK–c-Jun pathways were measured by Western blot. Activities of **d** NF-κB and **f** AP-1 were measured in rat liver nucleic protein by ^32^P-labeled gel-shift assay. Specificity of NF-κB (**e**) and AP-1 (**g**) were tested by incubating p50/p65 and c-Jun antibodies with nucleic protein, respectively. *Numerical* values represent the mean ratio and SEM of the optical density of phosphorylated form divided by total form of protein, or the optical density of radioactive bands (*n* = 7). Control group is set as 1.0. *C* control, *N* NAFLD, *S* SAMC, *N*+*S* NAFLD+SAMC. *Asterisk* represents *p* < 0.05 versus control group, while *Hash* represents *p* < 0.05 versus NAFLD group

To study the signaling pathway involved in the pathophysiological processes of NAFLD induced by the elevation of FFA and the effects of SAMC intervention, we measured the phosphorylation status and total expression of MAPK kinases signaling pathways including p38 MAPK, JNK and MEK/ERK, which play important roles in insulin and inflammatory responses [18, 19]. Interestingly, our result showed that an increase in the phosphorylation level of p38 MAPK and JNK/c-Jun, but a decrease in the phosphorylation of MEK/ERK1/2 was observed in NAFLD rat liver tissue. This finding suggested that the MAPK kinase pathways were differentially regulated during the development of NAFLD. Co-treatment of SAMC counteracted all the effects of NAFLD induction on the phosphorylation of these kinases without influencing the total expression levels except for total p38 MAPK where it was reduced by SAMC administration (Fig. 6a–c).

As critical regulators of cellular inflammation, proliferation and apoptosis, the activity of nuclear transcription factors NF-κB and AP-1 were induced in NAFLD rats. Co-treatment of SAMC during NAFLD induction significantly reduced the activity of both NF-κB and AP-1, while no obvious effect on the basal activity of these transcription factors was detected (Fig. 6d, f). The inhibitory effect of SAMC on the activity of NF-κB was through the reduction of the elevated phosphorylation of IκBα and recovery of degraded total IκBα in the cytosol (Fig. 6d). Supershift assay for NF-κB and AP-1 were used to confirm the specificities of these assays (Fig. 6e, g).

## Discussion

Common animal models that mimic the initiation and progression of NAFLD such as in dietary feeding and genetic manipulation have certain disadvantages. For example, a high-fat diet model lacks obvious steatohepatitis and genetic models (e.g. leptin, agouti, SREBP1c) often show decreased body weight, cellular fibrosis and inflammation [20]. The model used in the current study prevailed over such disadvantages by showing moderate levels of cellular fibrosis, oxidative stress, necroinflammation and significant increase in body weight (Supple Fig. 1A). Considering the fat composition in our diet recipe is closer to the clinical NAFLD fat level of about 30% when compared to other high-fat diet models (45–75%), the animal model used in the current study is better suited and is more physiological for studying the basic pathogenesis of NAFLD. Addition of SAMC effectively attenuated the pathological features of NAFLD in this model (e.g. hepatic injury, imbalance of lipid metabolism, fibrosis, oxidative stress and inflammation) without influencing the food intake of rats (Suppl. Fig. 1B), suggesting that the protective effects of SAMC were not simply from the reduction of food intake or loss of the body weight of rats, but may be from the direct or indirect actions of SAMC.

According to the “multi-hit” hypothesis, disrupted lipid metabolism and insulin resistance is the first step toward NAFLD development. Co-treatment with SAMC not only reduced the serum FFAs level that may induce the initial pathological changes during NAFLD [21], but also significantly lowered the lipid accumulation within the hepatocytes (Fig. 1). At the molecular level, this phenomenon was proven by the restoration of the lipid metabolism-related genes SREBP1c and adiponectin (Fig. 2). The mechanism whereby excessive FFAs in the blood induce insulin resistance is partly through the mediation of protein kinase C, resulting in impaired function of insulin receptor substrate-1 (IRS-1), which further activates JNK and SOCS3, contributing to insulin resistance [22–24]. In the current study, induced phosphorylation of JNK and up-regulated level of SOCS3 were reversed by the administration of SAMC, demonstrating the effectiveness of SAMC to suppress the process of FFAs-induced lipid imbalance, which might be one of the causal factors of insulin resistance (Figs. 3, 6).

In addition to the induction of hyperinsulinemia, excessive FFAs can also trigger the release of ROS and pro-inflammatory mediators from impaired liver cell organelles, thus contributing to the subsequent “hits” in the progression of NAFLD [25]. NAFLD development in our current model induced the expression of CYP2E1, which mediated the inhibition of antioxidant enzymes GPx and CAT, as well as the formation of lipid peroxidation end product (MDA) and NO metabolism end product (nitrotyrosine), leading to increased oxidative stress within the cell. Furthermore, increased inflammatory cytokines and chemokines, which are partly induced through the activation of NF-κB, further aggravated NAFLD by enhancing apoptosis and necrosis of the liver cells [26]. Since increased iNOS is an inducer of NO production, which takes part in cellular oxidative stress, there is also a positive feedback loop between oxidative stress and inflammation during the NAFLD development. In the current study, administration of SAMC reversed all these stimulatory processes and inhibitory effects of NAFLD on oxidative stress and inflammatory markers, which further demonstrated SAMC’s potent protective capability against NAFLD.

Molecules that initiate hepatic fibrosis (e.g. TGF-β_1_ and α-SMA) may also be activated by excessive FFAs through lipid peroxidation or cytokine production [26, 27]. In addition, activated hepatic Kupffer cells, the direct source of pro-inflammatory cytokine production, may in turn activate hepatic stellate cells (HSCs) to synthesize collagen, initiating the process of liver remodeling in the form of fibrosis and cirrhosis [28]. SAMC treatment not only attenuated the gene expression levels of TGF-β_1_ and α-SMA, but also reduced collagen accumulation around the centrilobular veins.

To further elaborate the molecular signaling pathways that link excessive FFAs and pathological events in NAFLD influenced by SAMC intervention, we evaluated the activation of MAPK kinases by phosphor-specific antibodies. It has recently been shown that the blockage of p38 MAPK in endothelial cells can prevent FFAs-induced insulin resistance through promoting the expression of PTEN gene or Akt phosphorylation [29]. Other reports also pointed out that the blockage of p38 MAPK accomplishes its anti-insulin resistance effect by decreasing both phosphorylation and basal expression of IRS1/2 genes [30, 31]. Here, we showed that the phosphorylation of p38 MAPK was significantly increased in NAFLD rat but decreased after the co-treatment of SAMC, suggesting the involvement of this kinase during NAFLD development and SAMC intervention. In addition, the role of JNK/c-Jun pathway in FFAs-induced insulin resistance is well studied. In the liver, JNK activity is increased in animal with insulin resistance. JNK1 knockout mice are resistance to high-fat-diet-induced insulin resistance [32]. Moreover, activation of JNK is known to promote inflammatory response and apoptosis in animal steatohepatitis model [33, 34]. Taken together, in this study, inhibition of the JNK/c-Jun phosphorylation by SAMC treatment not only attenuated the FFAs-induced insulin resistance, but also contributed to the reduction of necroinflammation during NAFLD. For the decrease in the phosphorylations of MEK1/2 and ERK1/2, our findings were consistent with a previous report conducted in a methionine-choline deficient (MCD) mice model [35]. The inhibition of MEK1/2/ERK1/2 and subsequent increased activity of transcription of AP-1 are responsible for hepatocyte sensitization to oxidative stress for cell death induction [35, 36]. Thus, the restoration of MEK1/2/ERK1/2 and inhibition of AP-1 activity by SAMC seem to be one of the important effects of SAMC when exerting its hepato-protective function during NAFLD. It is worth noting that when compared to the level of control group, SAMC treatment alone and SAMC + NAFLD co-treatment significantly increased the phosphorylation status of MEK1/2 and ERK1/2 (Fig. 6b). Since activation of MEK/ERK pathway is essential for the mediation of cell proliferation [37], the over phosphorylation of these kinases in the current study may imply the promotion of liver regeneration during NAFLD progression. Increased expression of liver regeneration marker gene, IL-6, further lends support to this hypothesis (Suppl. Fig. 2).

The activation of NF-κB during NAFLD relies mainly on the degradation of inhibitor of NF-κB, which is IκB. ROS and pro-inflammatory cytokines initiate the degradation of IκB [38]. Since NF-κB is the master regulator of molecules that take part in cellular proliferation, inflammation and apoptosis, then the inhibition of NF-κB by SAMC during NAFLD might be a critical step for the prevention of cascading inflammatory response and oxidative stress injury in the liver [5]. In this study, we clearly demonstrated that the inhibition of NF-κB by SAMC was through the recovery of cytosolic IκB expression (Fig. 6D).

In conclusion, we have, for the first time, reported the hepatoprotective effects of garlic-derived SAMC in a NAFLD rat model in vivo. Such protective effects are partly explained through the reduction on excessive FFAs-dependent pathways, fibrosis, oxidative stress, inflammation and diminished in the nuclear transcription factors (NF-κB and AP-1) activity during NAFLD development. MAPKs also mediated the attenuation of NAFLD pathological events by SAMC. However, the mediators between SAMC and FFAs has still need to be experimentally identified and further investigated. Expressional changes of FFAs, lipid metabolism genes and MAPKs indirectly indicated the possible improvement of insulin resistance from SAMC co-treatment. However, direct evidence from insulin tolerance test and glucose tolerance test on rats are essential to validate the possible beneficial effects of SAMC on insulin resistance. In addition, although the administration method used in the current study is intraperitoneal injection, previous study in human showed that oral administration of SAMC demonstrated its beneficial effect [39]. Actually, the dose of SAMC (200 mg/kg) used is not realistic for human consumption of aged garlic. However, a study in animal model within a relatively short period required a higher dose of SAMC in order to demonstrate the possible beneficial effects of SAMC. For human consumption, a lower dose with long-term consumption of aged garlic may provide similar protective effects against fatty liver disease [39]. Since garlic is a common food supplement around the world, we believe that garlic or its derivatives could be considered as one of the preventive measures in the treatment strategy of NAFLD.

## Electronic supplementary material

Below is the link to the electronic supplementary material.

**Supplement Figure 1.** Body weight change and weekly food intake of rats during NAFLD development. (A) Body weight of each rat was recorded every week. (B) high-fat diet food intake (ml/day) of each rat was recorded every day. Each dot represents the average food intake during one week. Data presented are expressed as Mean ± SEM (n = 7). (TIFF 65 kb)

**Supplement Figure 2.** IL-6 expression after NAFLD development with or without co-treatment of SAMC. (A) mRNA expression and (B) protein expression of IL-6 were measured by quantitative PCR or ELISA, respectively. Data presented are expressed as Mean ± SEM (n = 7), and experimental groups marked by different letters represented significant differences between groups at *p* < 0.05 (Kruskal–Wallis test followed by Dunn’s post hoc test). C: control; N: NAFLD; S: SAMC; N+S: NAFLD+SAMC. (TIFF 34 kb)
