# Supplementary figures and images for: Garlic-derived S-allylmercaptocysteine is a hepato-protective agent in non-alcoholic fatty liver disease in vivo animal model
Source: Eur J Nutr. 2012 Jan 26;52(1):179–91. doi: 10.1007/s00394-012-0301-0 (PMC3549410; doi:10.1007/s00394-012-0301-0)

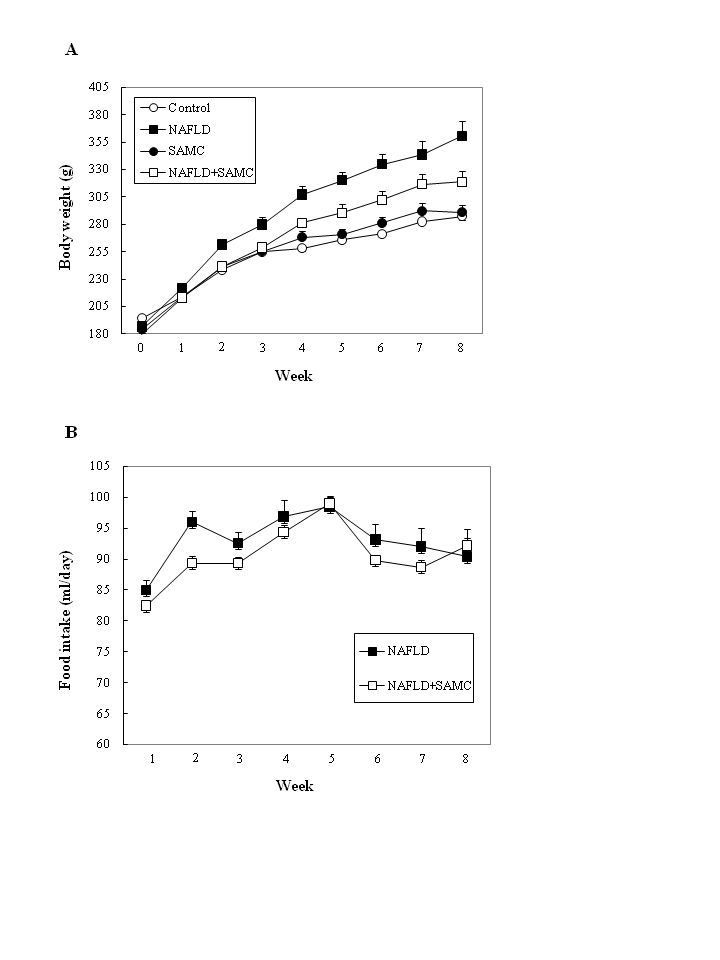

Supplement: Supplementary file 1 — Supplement Figure 1. Body weight change and weekly food intake of rats during NAFLD development. (A) Body weight of each rat was recorded every week. (B) high-fat diet food intake (ml/day) of each rat was recorded every day. Each dot represents the average food intake during one week. Data presented are expressed as Mean ± SEM (n = 7). (TIFF 65 kb) [file 394_2012_301_MOESM1_ESM.tif]

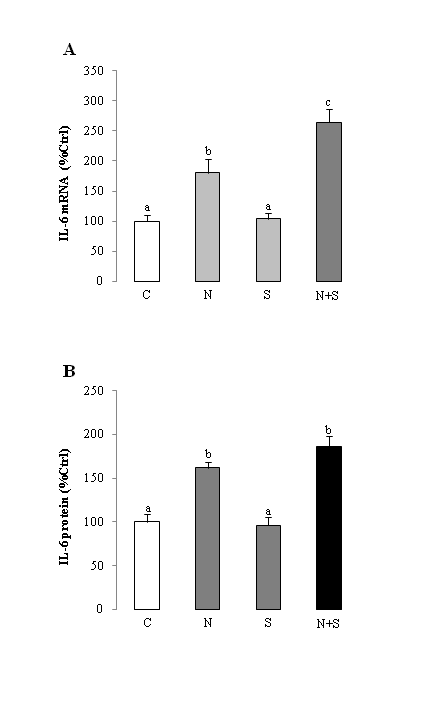

Supplement: Supplementary file 2 — Supplement Figure 2. IL-6 expression after NAFLD development with or without co-treatment of SAMC. (A) mRNA expression and (B) protein expression of IL-6 were measured by quantitative PCR or ELISA, respectively. Data presented are expressed as Mean ± SEM (n = 7), and experimental groups marked by different letters represented significant differences between groups at p < 0.05 (Kruskal–Wallis test followed by Dunn’s post hoc test). C: control; N: NAFLD; S: SAMC; N+S: NAFLD+SAMC. (TIFF 34 kb) [file 394_2012_301_MOESM2_ESM.tif]
